# The Prognostic Value of Troponin in Acute Pulmonary Embolism: A Systematic Review and Meta‐Analysis

**DOI:** 10.1002/ccd.70243

**Published:** 2025-10-21

**Authors:** Ahmed T. Elmewafy, James Waller, Priyanth Alaguraja, Kishan Desor, Ibrahim Antoun

**Affiliations:** ^1^ Department of Medicine The University of Sheffield Sheffield UK; ^2^ Department of Cardiology, Kettering General Hospital NHS Foundation Trust Kettering UK; ^3^ School of Medicine University of Leeds Leeds UK; ^4^ Department of Cardiovascular Sciences University of Leicester Leicester UK

**Keywords:** meta‐analysis, prognosis, pulmonary embolism, risk stratification, troponin

## Abstract

Acute pulmonary embolism (PE) is a major cause of cardiovascular morbidity and mortality. Troponin elevation is increasingly used for risk stratification, but its prognostic utility remains variably reported across studies. To evaluate the prognostic value of troponin elevation in patients with acute PE, concerning short‐term mortality and adverse clinical outcomes. A systematic review and meta‐analysis were conducted according to PRISMA 2020 guidelines. PubMed was searched from January 2000 to the present, using terms such as “pulmonary embolism,” “troponin,” and “prognosis.” Eligible studies reported associations between troponin elevation and mortality or adverse events in adult patients with PE. Data were synthesised quantitatively and narratively. The Quality in Prognosis Studies (QUIPS) tool was used to assess risk of bias. Sixty studies (*n *= 25,282) were included. Meta‐analysis showed that elevated troponin was significantly associated with increased in‐hospital mortality (OR: 5.42; 95% CI: 4.35–6.83), 30‐day mortality (OR: 4.35; 95% CI: 3.30–5.74), right ventricular dysfunction (OR: 3.42; 95% CI: 2.69–4.31), haemodynamic instability (OR: 3.29 95% CI: 2.48–4.39), and intensive care unit admission (OR: 5.81 95% CI: 3.52–9.68). Non‐meta‐analysed mortality data were similar to the meta‐analysed data, showing an association between elevated troponin levels and worse outcomes in PE. These associations were observed across both conventional and high‐sensitivity assays, as well as normotensive or low‐risk patients. Elevated troponin is a strong and consistent predictor of short‐term mortality and clinical deterioration in acute PE. With further research, it has the potential to be more widely integrated into risk stratification frameworks.

## 1 Introduction

Acute pulmonary embolism (PE) is a leading cause of cardiovascular morbidity and mortality [1], necessitating rapid and accurate risk stratification to guide management and improve outcomes. Prognostication in PE has advanced substantially with the integration of biomarkers, among which cardiac troponins—troponin I (cTnI) and troponin T (cTnT)—are of particular interest because of their sensitivity and specificity for myocardial injury. In the setting of acute PE, elevated troponin levels are believed to indicate right ventricular strain or injury as a consequence of the acute increase in pulmonary arterial pressure [2]. Multiple observational studies, prospective cohorts, and meta‐analyses have investigated the value of troponin as a prognostic marker in PE, leading to its incorporation into international guidelines and routine clinical practice [3, 4].

However, central questions remain regarding the precise role of troponin elevation in the prognosis of acute PE. First, there is an ongoing need to clarify whether troponin elevation is independently associated with adverse outcomes, such as short‐term mortality, major adverse cardiac events, and long‐term survival, especially when accounting for established clinical risk factors and imaging findings. Second, the consistency and strength of effect estimates for troponin's prognostic value vary across studies, reflecting differences in patient populations, PE severity, timing of measurement, and the use of various assay types. High‐sensitivity assays have enabled the detection of lower troponin concentrations. Still, this improved sensitivity may be offset by reduced specificity if inappropriate thresholds are used [3], necessitating further evaluation of optimal cutoffs, such as age‐ and context‐adjusted values, to maximize clinical utility.

Given this landscape, a comprehensive synthesis of the recent evidence is needed. This systematic review and meta‐analysis will combine data from recent studies to provide an updated quantitative evaluation of the prognostic value of troponin in acute PE. Specifically, this review will analyze whether troponin elevation, regardless of the assay type, independently predicts adverse outcomes in patients with PE, assess the consistency and magnitude of this effect, and compare the prognostic performance of traditional, contemporary, and highly sensitive troponin assays across different thresholds. The findings will inform clinicians about the utility and limitations of troponin as a risk stratification tool, helping to identify patients who may require closer monitoring, advanced therapies, or can be considered for early discharge.

By rigorously addressing these questions, this review aims to support the evidence‐based use of troponin measurements in the management of acute PE, refine risk stratification algorithms, and help standardise biomarker interpretation in diverse patient populations. Ultimately, this synthesis will facilitate more precise and individualized approaches to PE care, contribute to guideline development, and identify gaps in the literature where further research is required.

## 2 Methods

This systematic review followed the Preferred Reporting Items for Systematic Reviews and Meta‐Analyses (PRISMA) 2020 guidelines [5]. A protocol was registered in the International Prospective Register of Systematic Reviews (PROSPERO; ID: CRD420251075004) [6].

### 2.1 Study Eligibility

A detailed summary of our inclusion and exclusion criteria can be accessed by consulting our protocol, which is registered in the International Prospective Register of Systematic Reviews (PROSPERO; ID: CRD420251075004) [6].

### 2.2 Search Strategy

Systematic searches were conducted in PubMed from January 1, 2000, to the present. The search strategy employed a combination of Medical Subject Headings (MeSH) and free‐text keywords related to “pulmonary embolism,” “troponin,” “biomarkers,” “prognosis,” “mortality,” and other relevant clinical outcomes. No date restrictions were applied at the database search level; however, only studies published from the year 2000 onward were included during screening. Articles not published in English were excluded. The reference lists of all relevant systematic reviews and included studies were manually searched to identify any additional eligible studies.

All search results were imported into the Rayyan QCRI web application for initial screening and duplicate removal. Titles and abstracts were screened independently by two reviewers (J.W. and K.D.). Based on eligibility criteria, full texts of potentially eligible articles were then assessed for final inclusion. A third reviewer (A.E.) resolved any discrepancies during the selection process.

### 2.3 Data Extraction

Data was extracted using a standardised Excel sheet developed for this review. The following variables were collected: study title, authors, publication year, country, study design, sample size, population characteristics, and reported outcomes including mortality and adverse events (e.g., intensive care unit [ICU] admission, hypotension).

### 2.4 Study Outcomes

The primary outcomes were all‐cause in‐hospital mortality and 30‐day all‐cause mortality stratified by troponin elevation.

Secondary outcomes included associations between troponin levels and other adverse outcomes such as ICU admission, need for vasopressors, mechanical ventilation, and haemodynamic instability. Risk of bias was assessed using the Quality in Prognosis Studies (QUIPS) tool. Two reviewers independently evaluated each study (J.W. and A.E.), with discrepancies resolved by consensus or involvement of a third reviewer (P.A.). The certainty of evidence for each outcome was assessed using the GRADE (Grading of Recommendations, Assessment, Development and Evaluation) framework, considering study limitations, inconsistency, indirectness, imprecision, and publication bias. Outcomes were rated as high, moderate, low, or very low certainty. A Summary of Findings table was generated to present the results, and can be found in Table 2. We assessed potential publication bias using funnel plots and Egger's regression test, with *p* < 0.05 considered indicative of small‐study effects.

## 3 Results

A total of 534 references were identified through the database search. After removing duplicates and screening titles and abstracts, 65 studies met the eligibility criteria for full‐text review and final inclusion. Of these, five studies were excluded because the full text was not available in English. Thirty‐one studies contained sufficient data to be included in the quantitative synthesis. Specifically, 15 studies were included in the meta‐analysis assessing all‐cause in‐hospital mortality, and 16 studies contributed data to the meta‐analysis of 30‐day all‐cause mortality. The meta‐analysis was conducted using SPSS version 29.0.2. The results are presented in Table 1. The remaining studies, which did not report data suitable for meta‐analysis but were still relevant to the research question, were narratively synthesised to provide contextual insight into the prognostic value of troponin in acute PE. A summary of all included studies is presented in Table 1.

**Table 1:** A descriptive summary of all included studies.

Authors	Year	Country	Study setting	Sample size	Population description	Key findings
Australia						
Ng et al. [7]		Australia	Retrospective cohort study	577	Adult patients, including elderly, with confirmed PE, identified from hospital records and evaluated for prognosis of short‐term outcomes.	Cardiac troponin measurement, especially using high‐sensitivity assays, provided moderate accuracy for identifying higher‐risk PE patients who may benefit from increased monitoring or intervention.
Europe						
Giannitsis et al. [8]		Germany	Prospective cohort study	56	Hospitalized adult patients diagnosed with acute pulmonary embolism and followed for cardiac complications and mortality.	Elevated cTnT was significantly associated with increased risk of both early mortality and major adverse cardiac events in PE patients, underscoring its value in clinical risk assessment.
Pruszczyk et al. [9]		Poland	Prospective cohort study	64	Normotensive, initially stable adult patients (mean age 61), diagnosed with acute PE in a Polish hospital setting.	Elevated cTnT in normotensive patients with PE is a strong predictor of adverse clinical outcomes and in‐hospital mortality.
Kucher et al. [10]		Switzerland	Prospective cohort study	91	Adults with symptomatic and confirmed PE, systematically evaluated using biomarkers and echocardiography.	While cTnI measurements added most of the prognostic information for identifying high‐risk patients, a normal echocardiogram combined with a negative troponin I level was most useful to identify patients at the lowest risk for early death.
La Vecchia et al. [11]		Italy	Prospective observational study	48	Consecutive adult patients with confirmed acute PE, requiring admission to the coronary care unit, and without known coronary artery disease.	Elevated cTnI on admission is a frequent finding in acute PE patients requiring intensive care and is the strongest independent predictor of in‐hospital mortality; even modest increases suggest increased risk, highlighting troponin's role in risk stratification for this population.
Kostrubiec et al. [12]		Poland	Prospective study	100	Consecutive patients admitted to the Department of Internal Medicine at the Medical University of Warsaw between 1999 and 2004, with PE confirmed by thrombo‐emboli visualization in at least segmental arteries by contrast‐enhanced spiral computed tomography or by high probability lung scintigraphy.	Simultaneous measurement of serum cTnT and NT‐proBNP allows for precise APE prognosis. Normotensive patients on admission with cTnT 0.07 mg/L and NT‐proBNP 600 ng/L are at high risk of PE mortality, whereas NTproBNP 600 ng/L indicates excellent prognosis.
Aksay et al. [13]		Turkey	Retrospective chart review	77	Adult patients with confirmed acute PE who had cTnI levels measured at admission to the emergency department.	Elevated cTnI levels in acute pulmonary embolism are significantly associated with higher rates of in‐hospital mortality and adverse clinical outcomes, underscoring the value of cTnI as a useful marker for early risk stratification and guiding management decisions in the emergency department.
Maziere et al. [14]		France	Prospective, blinded study	60	Adult outpatients diagnosed with acute PE in an emergency department in Paris, France, mainly elderly (mean age 72 years), half above 75 years.	cTnI did not significantly differentiate between PE patients who died and those who did not, as its mean value showed no statistically significant difference between complicated and noncomplicated cases (*p* = 0.93)
Puls et al. [15]		Germany	Prospective observational study	107	Adults admitted with confirmed acute PE to a German university hospital, predominantly female, with a mean age of 61 years.	Elevated cTnT on admission was associated with significantly higher 30‐day mortality rates. Although elevated admission troponin predicted increased mortality, its positive predictive value (PPV) for mortality was lower compared to H‐FABP. In multivariable analysis, troponin T was not an independent predictor of overall mortality, unlike H‐FABP.
Gallotta et al. [16]		Italy	Prospective, non‐randomised, open study	90	Consecutive adult patients with central sub‐massive or non‐massive PE, haemodynamically stable at admission, confirmed by spiral CT, with appropriate exclusions (e.g., acute/chronic renal failure, recent myocardial infarction, initial haemodynamic instability).	A mild increase in cTnI on admission independently predicts the risk of developing haemodynamic instability during hospitalization in patients with central sub‐massive or non‐massive PR. Higher continuous troponin values also trend with in‐hospital death risk, though not independently of all covariates.
de Bonis et al. [17]		Italy	Retrospective case‐control study	67	Elderly patients (≥ 65 years) admitted with massive PE and treated with thrombolysis.	In elderly patients with massive PE, high serum cTnI at admission and the development of thrombocytopenia after thrombolysis are strong predictors of in‐hospital mortality, while a history of cancer or cardiovascular disease predicts higher long‐term mortality.
Jiménez et al. [3]		Spain	Prospective cohort study	318	Haemodynamically stable adults with objectively confirmed acute symptomatic PE presenting to a single Spanish emergency department.	In stable acute PE patients, elevated cTnI was not predictive of 30‐day all‐cause mortality but did independently predict risk for fatal pulmonary embolism. The high negative predictive value of cTnI suggests it is useful for identifying patients at lower risk for PE‐related death, but it is less helpful alone for ruling out all‐cause mortality.
Bova et al. [18]		Italy	Prospective, observational cohort study	201	Consecutive adult patients with confirmed acute PE, who were normotensive at baseline and treated with anticoagulation across six Italian centers.	Commonly used risk stratification tools, including cTnI, are not useful predictors of in‐hospital PE‐related adverse events among haemodynamically stable patients; however, clinical score and cTnI are independent predictors of 3‐month all‐cause mortality. Aggressive therapy based on these markers in stable patients may be unwarranted.
Janata et al. [19]		Austria	Retrospective cohort study	737 total; 563 with TNT measured	Consecutive adult patients diagnosed with proven acute pulmonary embolism at a single tertiary center, with cTnT measured at initial emergency department presentation.	cTnT‐positive patients had significantly higher in‐hospital and 1‐year mortality rates than TNT‐negative patients.
Vuilleumier et al. [20]		Switzerland, France, Belgium	Prospective cohort study	146	Consecutive patients presenting at the emergency departments of three primary to tertiary care university hospitals (Geneva, Switzerland; Brest, France; and Saint Luc, Bruxelles, Belgium) with clinically suspected acute PE were screened.	Of the cardiac biomarkers studied, NT‐proBNP was the most powerful independent predictor of adverse outcomes in patients with non‐massive PE.
Moores et al. [21]		Spain	Prospective cohort study	567	Consecutive adults with objectively confirmed, symptomatic, haemodynamically stable acute PE presenting to a single Spanish center's emergency department.	The pulmonary embolism severity index (PESI) is more accurate than troponin I for identifying low‐risk patients with acute PE. Adding troponin testing does not improve risk stratification over PESI alone.
Kostrubiec et al. [22]		Poland	Prospective cohort study	220	220 consecutive patients with PE, 86 males and 134 females, aged 64 ± 18 years, in Warsaw, Poland.	Impaired kidney function, present in 47% of PE patients, is related to all‐cause mortality. In initially normotensive patients, a GFR < 35 mL min−1 predicts 30‐day mortality. Moreover, GFR assessment can improve troponin‐based risk stratification of PE.
Lankeit et al. [23]		Germany	Prospective cohort study	156	Consecutive patients who were diagnosed with acute PE at the Universities of Göttingen and Heidelberg between March 2007 and April 2009, and gave informed consent.	Hs‐cTnT assays may be capable of improving risk stratification of non‐high‐risk PE.
Ozsu et al. [24]		Turkey	Prospective cohort study	108	Consecutive normotensive adult patients with acute PE confirmed by CTPA and managed in a tertiary referral hospital in Turkey.	In normotensive PE, the combination of NT‐proBNP and cTnT is a stronger predictor of 30‐day all‐cause mortality than right ventricular dysfunction assessed by CTPA or echocardiography, making multimarker strategies based on biomarkers more valuable for risk stratification.
Kukla et al. [25]		Poland	Prospective cohort study	225	Patients aged from 17 to 89 years old diagnosed with acute PE.	ECG changes linked to elevated troponin in APE: S1Q3T3, T‐wave inversion (V2–V4), ST depression (V4–V6), ST elevation (III, V1), QR in V1, widespread T‐wave inversions. ECG predictors of in‐hospital death: S1Q3T3, QR in V1, ST depression (V4–V6), ST elevation (III, V1).
Lankeit et al. [26]		Germany, Spain, Poland	Prospective cohort study	526	Adult, haemodynamically stable patients presenting with acute, objectively confirmed PE.	Both hsTnT (cutoff 14 pg/mL) and the sPESI reliably identify increased risk for adverse outcomes and mortality after acute PE. Their combination identifies a very low‐risk subset with excellent short‐ and long‐term prognosis, and these patients may be considered for early discharge or outpatient management.
Singanayagam et al. [27]		UK	Retrospective cohort study	411	Normotensive adults with CT‐confirmed, community‐onset acute PE; excluded if inpatient PE, hypotensive, or incomplete biomarker data.	The PESI score is more accurate than biomarkers alone for predicting 30‐day mortality after acute PE, but the addition of cTnI to PESI improves risk prediction. d‐dimer is associated with higher mortality but does not independently add prognostic value beyond troponin or PESI.
Jiménez et al. [28]		Spain	Prospective cohort study	591	Consecutive normotensive adults with objectively confirmed acute symptomatic PE presenting to an emergency department in Spain.	Combining either troponin or echocardiography with CCUS improves the identification of high‐risk normotensive patients with acute symptomatic PE—beyond single‐test approaches—and more effectively predicts 30‐day PE‐related mortality.
Spirk et al. [29]		Switzerland	Prospective cohort study	369	Adults (aged ≥ 18 years) with objectively confirmed acute PE diagnosed at 18 Swiss hospitals, including both inpatients and outpatients.	Cardiac troponin testing provides significant incremental prognostic value for identifying early death and recurrent PE in patients with a high sPESI, but is not required for low sPESI individuals; combining sPESI with troponin results in superior risk stratification compared to sPESI alone.
Labyk et al. [30]		Poland	Retrospective cohort study	353	Consecutive patients hospitalized with PE (mean age 64.7 years, 141 males, 212 females), predominantly diagnosed by CT in a referral hospital, with comprehensive comorbidity data captured.	Clinical and biomarker‐based risk stratification is crucial for prognosis in acute PE. cTnI, creatinine, and NT‐proBNP are significant predictors for mortality and adverse events.
Thielmann et al. [31]		Germany	Retrospective cohort study	46	All patients had a confirmed diagnosis of acute PE and underwent acute surgical pulmonary embolectomy at a single tertiary care center in Germany.	Preoperative cTnI elevation on admission is an independent predictor of increased in‐hospital mortality and major adverse clinical events following surgical intervention for PE.
Apfaltrer et al. [32]		Germany	Retrospective observational study	65	Adults who were referred to the hospital's chest pain unit with clinical suspicion of acute PE, had PE confirmed by CTPA, and had their hs‐cTnI serum levels measured during admission.	Hs‐cTnI is associated with adverse clinical outcomes in patients with acute pulmonary embolism. Combining hs‐cTnI measurements with quantitative CT parameters improves the accuracy of predicting which patients are at higher risk for poor outcomes, such as intensive care treatment or death.
Becattini et al. [33]		Italy	Prospective cohort study	1716	Adults (aged 18 years and older) with objectively confirmed acute PE who were admitted to academic or community hospitals throughout Italy and enrolled in the Italian Pulmonary Embolism Registry.	In haemodynamically stable patients with acute pulmonary embolism, using an integrated risk stratification model that combines both right ventricular dysfunction (assessed by echocardiography) and myocardial injury (assessed by troponin levels) offers improved prognostic accuracy for in‐hospital death or clinical deterioration compared to using either marker alone.
Kukla et al. [34]		Poland	Retrospective cohort study	500	Individuals with a confirmed diagnosis of PE who were hospitalized in seven community hospital cardiology departments in Poland between 2005 and 2012.	In patients with PE especially those classified as intermediate risk—ischemic ECG patterns on hospital admission (particularly the ST‐segment ischemic pattern, STIP) are independent predictors of worse in‐hospital outcomes, including higher rates of complications and death.
Babaoglu et al. [35]		Turkey	Retrospective observational study	98	The population assessed in this study consists of 98 adult patients diagnosed with PE who were referred to Ankara Ataturk Training and Research Hospital between June 2009 and July 2011.	Certain hepatic, cardiac, and renal biomarkers—such as lactate dehydrogenase, creatinine, uric acid, cTnI, NT‐proBNP, CK‐MB, d‐dimer, and ESR—differ significantly among high‐, intermediate‐, and low‐risk pulmonary thromboembolism (PTE) patients. In emergency situations where echocardiography is not available, elevated levels of these biomarkers can help identify patients at higher risk of mortality.
Akgüllü et al. [36]		Turkey	Retrospective cohort study	288	The study population consisted of adult patients diagnosed with acute PE who were hospitalized at Adnan Menderes University Faculty Hospital in Aydın, Turkey, between January 2011 and April 2013.	A combination of easily obtainable clinical and laboratory markers—including cTnII, creatinine, mean platelet volume (MPV), neutrophil to lymphocyte ratio, corrected QT interval dispersion, and P wave dispersion—can more accurately predict early (in‐hospital) death in patients with acute PE than the traditional sPESI scoring system alone.
Kaeberich et al. [37]		Germany, Poland, Spain	Prospective cohort study	662	Normotensive adults with confirmed acute PE from multiple European centers.	Age‐adjusted hsTnT cut‐off values (14 pg/mL for < 75 years, 45 pg/mL for ≥ 75 years) improve risk stratification in normotensive pulmonary embolism, offering additive prognostic value beyond standard clinical scores and echocardiography for prediction of adverse 30‐day outcomes; a three‐step strategy using sPESI, hsTnT, and RV dysfunction identifies high‐risk patients who may require advanced therapy.
Langer et al. [38]		Germany	Prospective observational study	161	Consecutive normotensive adult patients with confirmed PE admitted to a German university hospital.	H‐FABP and CK‐MB are strong, independent predictors of 30‐day mortality in normotensive PE patients and outperform cTnI in risk stratification; cTnI shows a weak association with mortality and is less useful in this intermediate‐risk group.
Ozsu et al. [39]		Turkey	Retrospective cohort study	489	Normotensive adults with confirmed acute symptomatic PE, excluding those with hypotension or shock, admitted to a tertiary care setting.	The combination of elevated cardiac troponin and shock index ≥ 1 can effectively identify normotensive acute PE patients at higher risk of 30‐day mortality, providing a simple clinical tool for intermediate risk stratification and better clinical decision‐making. External validation is needed before clinical implementation.
Granot et al. [40]		Israel	Retrospective cohort study	370	Adult inpatients (≥ 18 years) with acute pulmonary embolism confirmed by CTPA and with troponin testing within 24 h of CTPA, from a university‐affiliated hospital in Israel.	CT‐derived cardiac chamber volumes, particularly the right ventricular to left ventricular volume ratio (VVR), correlate strongly with troponin elevation in acute PE. Higher VVR values not only are predictive of myocardial injury (as detected by troponin) but also are associated with significantly increased short‐term mortality risk.
Cennamo et al. [41]		Italy	One retrospective phase, one prospective phase	374	Adults (> 18 years) presenting to the emergency department with confirmed acute PE, excluding those with acute coronary syndrome.	Combining hs‐cTnI levels with the PESI score is more effective than the PESI score alone for risk stratification of acute PE patients in the emergency department, improving decisions about safe discharge versus the need for intensive care.
Sonne‐Holm et al. [42]		Denmark	Retrospective cohort study	563	Unselected Danish adults with first‐time, hospital‐diagnosed PE and troponin measurement available.	Higher troponin concentrations, not just values above the reference threshold, are strongly associated with increased 30‐day mortality in acute PE.
Boris et al. [43]		Serbia	Retrospective cohort study	1677	Patients with confirmed acute PE admitted to seven regional hospitals and treated according to current guidelines; includes both sexes, all adult ages, and varied comorbidities.	Elevated BNP (relative to upper normal limit), like troponin, significantly predicts all‐cause and PE‐related 30‐day mortality in acute PE; BNP may enhance early mortality risk stratification, especially for intermediate‐high‐risk cases.
Naum et al. [44]		Romania	Retrospective cross‐sectional study	109	109 adults (over 18 years of age) who were hospitalized and treated for acute pulmonary embolism at “St. Spiridon” Emergency Hospital, Iasi, between February 2021 and August 2022.	Laboratory biomarkers—specifically d‐dimer and cTnT, are valuable predictors for 7‐day mortality in PE patients overall and in key subgroups (atrial fibrillation, COVID‐19, and cancer).
Sonne‐Holm et al. [45]		Denmark	Retrospective cohort study	1539	Danish PE patients (≥ 18 years old) from 2013 to 2018 from a national registry.	The study found that early serial troponin I/T measurements reveal distinct risk patterns in PE patients, with a rapid rise within 10 h linked to the highest 30‐day mortality.
Asia						
Te Hsu et al. [46]		Taiwan	Retrospective cohort study	110	Adult patients with confirmed acute PE from a single center in Taiwan.	The combination of elevated troponin I and RVD is a strong predictor of 100‐day mortality in acute PE, identifying patients who may benefit most from aggressive therapy such as thrombolysis.
Zhu et al. [47]		China	Prospective cohort study	90	Confirmed cases of acute PE, age ≤ 75 years, presenting within 30 days from onset, from 12 hospitals in China.	Both RVD and elevated cTnI are independent predictors of poor 14‐day outcomes in acute pulmonary embolism.
Ng et al. [48]		Hong Kong	Retrospective, observational cohort study	100	Consecutive Chinese adults admitted for acute PE at a regional Hong Kong hospital between January 2002 and December 2009.	Elevated cTnI levels are predictive of haemodynamic instability and complicated clinical course in Chinese patients with acute PE, cTnI was not significantly associated with all‐cause in‐hospital mortality.
Choi et al. [49]		South Korea	Retrospective cohort study	657	Hospitalized adult patients with a CT‐confirmed diagnosis of acute PE at a single tertiary center in Korea between 2003 and 2012.	Computed tomographic right ventricular dilation is an independent prognostic marker of adverse outcomes in acute pulmonary embolism. Its prognostic value increases when combined with established markers such as PESI, NT‐proBNP, and troponin I.
Tanabe et al. [50]		Japan	Retrospective cohort study	441	Consecutive patients admitted for acute PE to participating Tokyo CCUs between 2009 and 2011.	Blood biomarkers—specifically BNP, troponin, and blood glucose levels—can predict 30‐day mortality in Japanese patients with acute pulmonary embolism; among these, elevated troponin is significantly associated with higher short‐term mortality.
Ghaffari et al. [51]		Iran	Prospective cohort study	627	Hospitalized adults aged 18–85 years with confirmed acute PE presenting within 24 h of symptom onset, with follow‐up throughout hospitalization and up to 1 year.	cTnI elevation is an independent and clinically meaningful predictor of adverse short‐term outcomes, including mortality and major adverse cardiopulmonary events, in acute PTE. Routine cTnI measurement can aid risk stratification and clinical decision‐making in this setting.
Kim et al. [52]		South Korea	Retrospective cohort study	374	The study assesses patients diagnosed with acute PE. A total of 374 patients were included in the analysis, after excluding those with end‐stage renal disease requiring hemodialysis or missing hsTnT levels.	A hs‐cTnT level of 60 ng/L serves as a valuable prognostic marker in patients with acute PE. Patients exceeding this threshold demonstrated a higher risk of both PE‐related adverse outcomes and all‐cause mortality within 30 days.
Jiao et al. [53]		China, Japan	Retrospective cohort study	213	Normotensive patients (defined as a SBP > 90 mmHg) hospitalized for acute PE in China‐Japan Friendship Hospital from January 2017 to February 2020. Only adult patients with objectively confirmed PE and no reperfusion procedures at the time of PE diagnosis were enrolled.	ECG abnormalities and biomarkers on admission may provide a rapid and effective approach to identify patients with poor prognoses during hospitalization.
South America						
Seropian et al. [54]		Argentina	Retrospective cohort study	73	Hospitalized adults with intermediate or high‐risk PE and discordant RV serum biomarker profiles (either high hs‐cTnT/low NT‐proBNP or vice versa), admitted to a tertiary care center in Argentina.	Among patients with intermediate or high‐risk PE and discordant RV strain biomarkers, those with high troponin but low NT‐proBNP have significantly worse short‐term outcomes (higher rates of death, cardiac arrest, mechanical ventilation, or inotrope use) than those with the opposite discordance (“high BNP discordance”).
North America						
Konstantinides et al. [55]		USA	Prospective, observational cohort study	106	Consecutive adult patients with acute PE confirmed by imaging, primarily women (61 average age).	Cardiac troponins I and T, particularly when markedly elevated, reliably identify patients with acute PE at higher risk of mortality and major complications; their measurement enables improved risk stratification and may aid in guiding management decisions.
Mehta et al. [56]		USA	Retrospective cohort study	38	Consecutive patients with acute PE who were treated in Long Island College Hospital between January 1998 and December 2000 were reviewed retrospectively.	Patients with acute PE with elevated serum cTnI levels are at a higher risk for the development of right ventricular dysfunction and cardiogenic shock. Serum cTnI has a role in risk stratification and short‐term prognostication in patients with acute pulmonary embolism.
Yalamanchili et al. [57]		USA	Retrospective cohort study	741 (147 with PE)	The 147 patients with acute PE included 73 men and 74 women (mean age 58 ± 16 years; range 19−99).	cTnI levels were increased in 24 of 147 patients (16%) with documented acute pulmonary embolism and in 20 of 594 patients (3%) without pulmonary embolism (*p* < 0.001). In patients with acute pulmonary embolisms, 8 of 24 (33%) with increased cardiac troponin I levels and 9 of 123 (7%) with normal cardiac troponin I levels died during hospitalization (*p* < 0.001).
Scridon et al. [58]		USA	Retrospective cohort study	141	Adults diagnosed with acute PE at a single academic center in the United States, with available echocardiography and troponin data.	The combination of elevated cTnI and right ventricular enlargement identifies acute PE patients at greatly increased risk of 30‐day mortality—up to seven times that of patients with neither marker.
Douketis et al. [58]		Canada, Netherlands	Prospective cohort study	458	Adult patients with objectively confirmed, acute, symptomatic, submassive PE, excluding those with massive PE or life expectancy < 3 months. Both sexes and a wide age range were included (19–92 years).	In patients with submassive PE, about 1 in 7 have elevated cTnI, which is associated with a 3.5‐fold higher risk of all‐cause death but not with increased risk of recurrent venous thromboembolism.
Kline et al. [60]		USA	Prospective cohort study	152	Consecutive normotensive patients with acute PE diagnosed by CT angiography, excluding those with high short‐term mortality risk or conditions preventing follow‐up/assessment.	Of eight evaluated biomarkers, only BNP and cTnI were predictive of RVD after submassive PE, with BNP being a stronger predictor of both mortality and exercise intolerance. cTnI had only modest, nonsignificant predictive power for mortality and adverse outcomes in this setting.
Stein et al. [61]		USA	Retrospective cohort study	1273	Hospitalized, normotensive adult patients with acute PE, excluding those with haemodynamic instability or significant comorbidities affecting RV size.	Elevated cTnI levels and right ventricular enlargement, alone and especially in combination, are associated with increased in‐hospital mortality and all‐cause mortality in stable (normotensive) patients with acute PE, but the risk is not high enough to justify routine use of thrombolytic therapy.
Chughtai et al. [62]		USA	Retrospective cohort study	78	Adult hospitalized patients with massive PE treated with thrombolytic therapy in Michigan hospitals from 2000 to 2010.	Shock is the only significant independent predictor of in‐hospital mortality in patients with massive PE receiving thrombolytic therapy, but the combination of shock with high cTnI and RVD or enlargement is associated with the highest mortality risk.
Hakemi et al. [63]		USA	Retrospective cohort study	298	Adults hospitalized with confirmed acute PE at a tertiary hospital in Chicago.	An undetectable highly sensitive cTnI identifies patients with acute PE at extremely low risk for in‐hospital mortality and other major adverse outcomes, supporting its use for safely triaging selected patients to outpatient management.
Lee Chuy et al. [64]		USA	Retrospective cohort study	289	Adult patients with confirmed acute pulmonary embolism and cTnI measured during initial hospitalization.	Detectable highly sensitive cTnI predicts higher long‐term mortality in patients with acute PE, especially among those classified as low risk by PESI. Patients with low‐risk PESI and elevated cTnI may require closer follow‐up after discharge.
Goraya et al. [65]		USA	Retrospective cohort study	314	Adults diagnosed with acute, haemodynamically stable PE in the ED at a US academic medical center; included only those with hs‐TnT measured.	Distinct high‐sensitivity troponin T cutoffs optimize risk stratification for acute PE: 12 pg/mL identifies patients at low risk for short‐term adverse outcomes, while 87 pg/mL is optimal for detecting increased risk of mortality and ICU admission in higher‐risk patients based on clinical and imaging criteria.

Abbreviations: BNP, brain natriuretic peptide; CCUS, critical care ultrasound; CK‐MB, creatine kinase‐MB; CT, computed tomography; cTnI, cardiac troponin I; cTnT, cardiac troponin T; CTPA, computed tomography pulmonary angiogram; H‐FABP, heart‐fatty acid binding protein; hs‐cTnT, high sensitivity cardiac troponin T; hs‐cTNI, high sensitivity cardiac troponin I; NT‐proBNP, N‐terminal prohormone of brain natriuretic peptide; PE, pulmonary embolism; RVD, right ventricular dysfunction; sPESI, stratified pulmonary embolism severity index.

### 3.1 Risk of Bias Assessments

**Figure 1:**
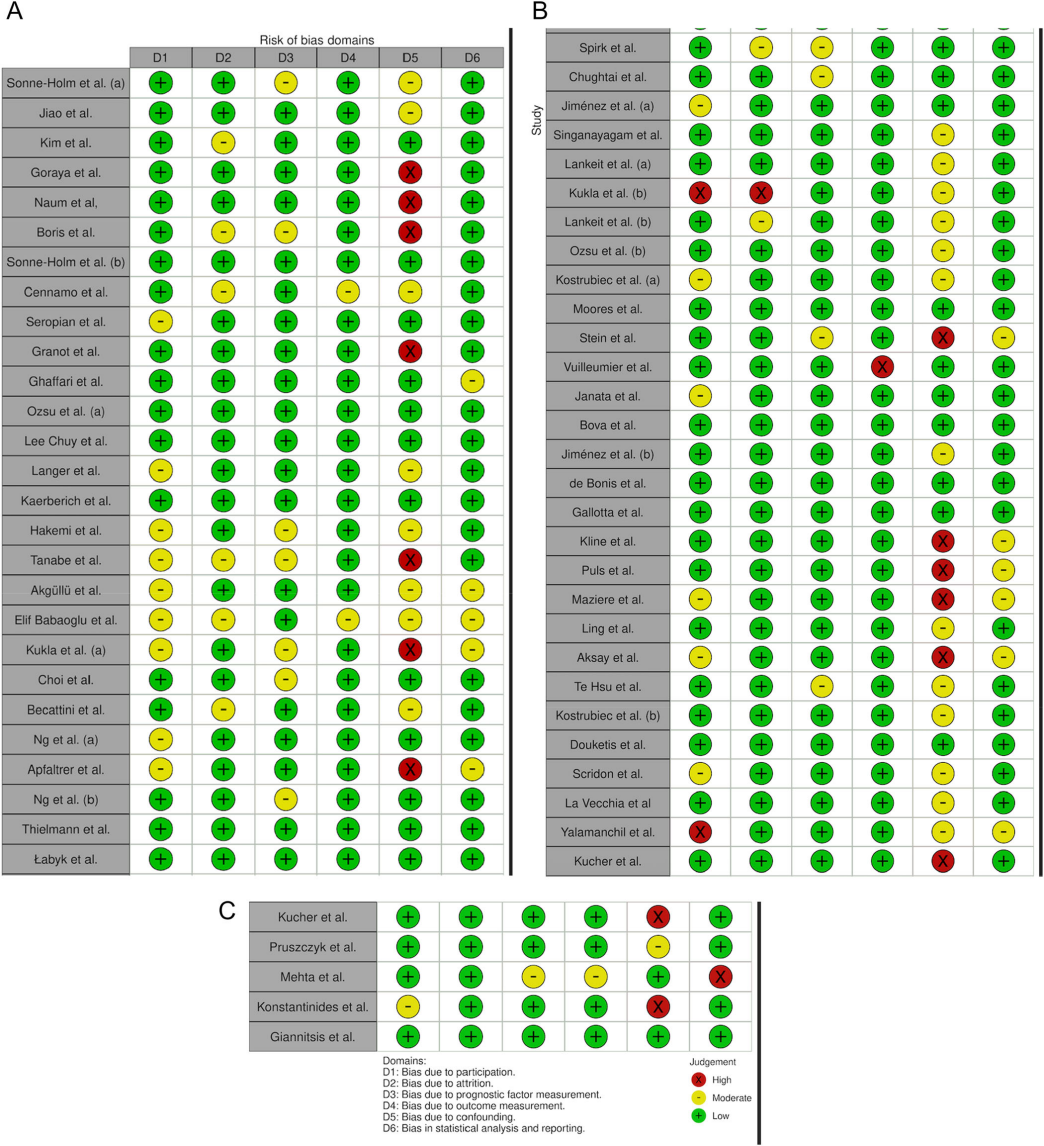
A diagram [70] to illustrate the predicted bias of non‐randomised studies included in this review (A–C). [Color figure can be viewed at wileyonlinelibrary.com]

The risk of bias assessment, summarized in Figures 1, shows that most studies had low to moderate risk across key QUIPS domains. Selection bias and outcome measurement were generally well addressed, while confounding was the most common area of concern (Central illustration 1).

**Central illustration:**
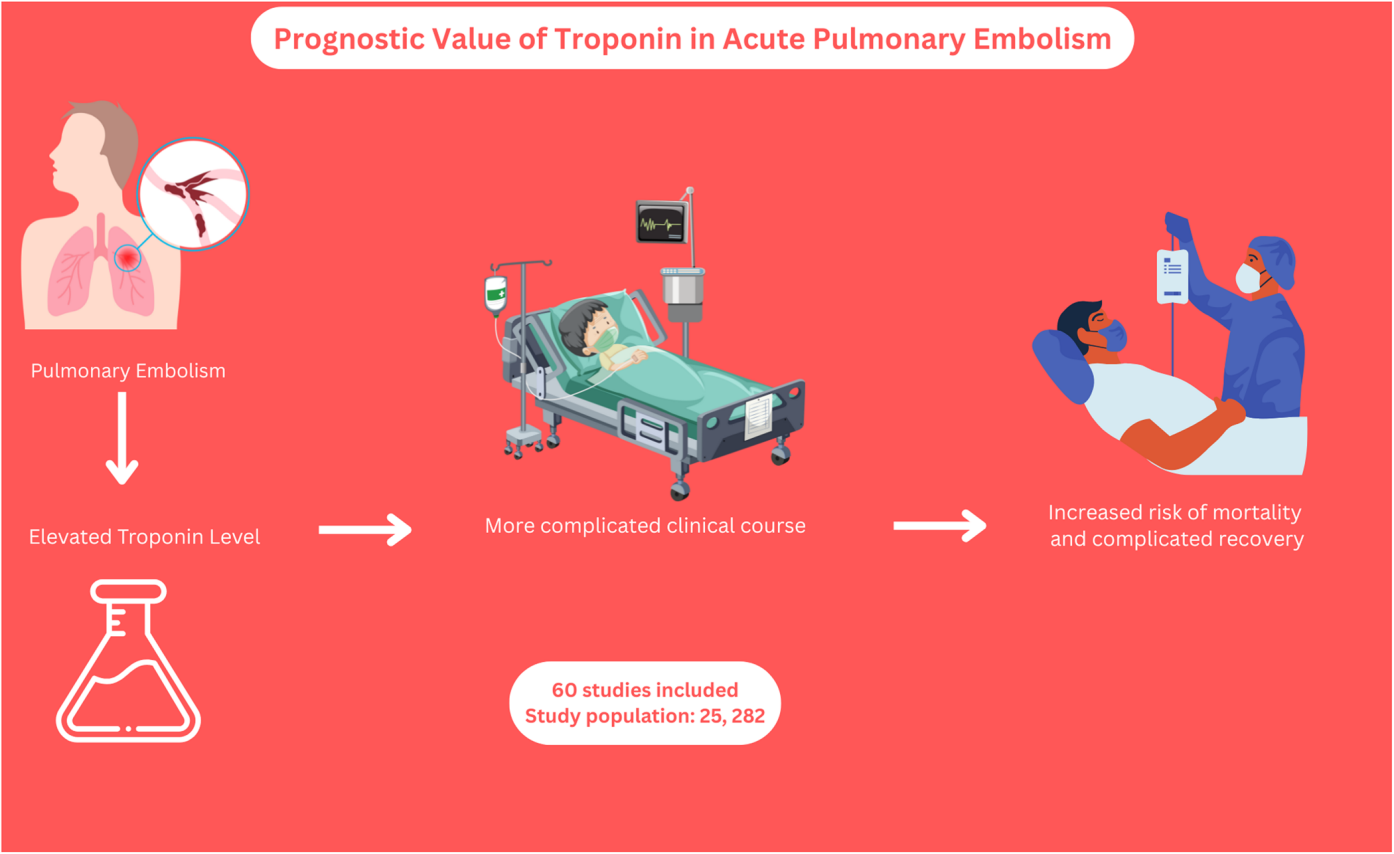
Conceptual framework for troponin as a biomarker in acute pulmonary embolism wileyonlinelibrary.com].

### 3.2 Quantitative Synthesis

A total of 15 studies reported data on the association between troponin levels and in‐hospital all‐cause mortality. The forest plot in Supporting Information S1: Figure S1 illustrates this relationship; the pooled analysis demonstrated a statistically significant odds ratio of 4.35 (95% CI: 3.30–5.74), with no observed heterogeneity (*I*² = 0%), indicating a strong and consistent association. Egger's regression test demonstrated significant funnel plot asymmetry (intercept = 1.09, 95% CI: 0.50–1.68, *p* = 0.002), suggesting the presence of small‐study effects and possible publication bias. This was visually supported by inspection of the funnel plot (Supporting Information S1: Figure S6). The certainty of evidence for this outcome was rated as high according to the GRADE framework (Table 2).

**Table 2:** Summary of findings (GRADE) for the association between elevated troponin and adverse outcomes in acute pulmonary embolism.

Outcome	No of studies	Effect (95% CI)	Certainty of evidence (GRADE)	Notes
In‐hospital mortality	15	OR 5.42 (4.35–6.83)	●●●● High	Consistent effect, large magnitude
30‐day mortality	16	OR 4.35 (3.30–5.74)	●●●● High	Robust findings, narrow CI
Right ventricular dysfunction (RVD)	21	OR 3.42 (2.69–4.31)	●●●◯ Moderate	Downgraded for heterogeneity in RVD definitions
Haemodynamic instability	12	OR 3.29 (2.48–4.39)	●●●● High	Consistent results, precise estimates
ICU admission	4	OR 5.81 (3.52–9.68)	●●●◯ Moderate	Downgraded due to the limited number of studies

A total of 16 studies reported data on the association between troponin levels and 30‐day all‐cause mortality. The forest plot in Supporting Information S1: Figure S2 illustrates this relationship, with the pooled analysis yielding a statistically significant odds ratio of 5.42 (95% CI: 4.35–6.83), with low heterogeneity (*I*² = 11%). Egger's regression test indicated significant funnel plot asymmetry (intercept = 1.24, 95% CI: 0.90–1.58, *p* < 0.001), with smaller studies tending to report stronger effects (slope = 1.24, *p* = 0.011). This finding was consistent with the visual appearance of the funnel plot (Supporting Information S1: Figure S7). The certainty of evidence for this outcome was rated as high according to the GRADE framework (Table 2).

A total of 21 studies reported data on the association between troponin elevation and right ventricular dysfunction (RVD). The forest plot in Supporting Information S1: Figure S3 illustrates this relationship; the pooled analysis demonstrated a statistically significant odds ratio of 3.42 (95% CI: 2.69–4.31). Heterogeneity was moderate (*I*² = 50%), indicating some variability in effect estimates across studies. Egger's regression test did not demonstrate significant funnel plot asymmetry (intercept = 0.53, 95% CI: –0.32 to 1.37, *p*= 0.21), indicating no clear evidence of publication bias, consistent with the symmetric appearance of the funnel plot (Supporting Information S1: Figure S8). The certainty of evidence for this outcome was rated as moderate according to the GRADE framework (Table 2).

A total of 12 studies reported data on the association between troponin elevation and haemodynamic instability. The forest plot in Supporting Information S1: Figure S4 illustrates this relationship; the pooled analysis demonstrated a statistically significant odds ratio of 3.29 (95% CI: 2.48–4.39). No heterogeneity was observed (*I*² = 0%), indicating a consistent association across studies. Egger's regression test was not statistically significant (intercept = 0.63, 95% CI: –0.20 to 1.45, *p* = 0.12), suggesting no evidence of publication bias, which was supported by visual inspection of the funnel plot (Supporting Information S1: Figure S9). The certainty of evidence for this outcome was rated as high according to the GRADE framework (Table 2).

A total of four studies reported data on the association between troponin elevation and ICU admission. The forest plot in Supporting Information S1: Figure S5 illustrates this relationship; the pooled analysis demonstrated a statistically significant odds ratio of 5.81 (95% CI: 3.52–9.68). Heterogeneity was minimal (*I*² = 0%), indicating a consistent effect across studies. Visual inspection of the funnel plot (Supporting Information S1: Figure S10) suggested no major evidence of publication bias. Egger's regression test did not show significant funnel plot asymmetry (intercept = 0.98, 95% CI: –1.34 to 3.29, *p* = 0.31), indicating no evidence of publication bias for this outcome. The funnel plot appeared symmetric (Supporting Information S1: Figure S10). The certainty of evidence for this outcome was rated as moderate according to the GRADE framework (Table 2).

### 3.3 Mortality in Non‐Meta‐Analysed Studies

Elevated troponin is generally associated with increased mortality in patients with acute PE, as demonstrated across multiple studies excluded from the meta‐analysis. For example, Ghaffari et al. reported that patients with elevated cTnI had significantly higher in‐hospital mortality compared to those with negative cTnI (22.3% vs. 8.6%, *p* < 0.001) [51]. Additionally, the prognostic value of troponin in normotensive or “low‐risk” patients with PE was demonstrated by Kaeberich et al., who found that elevated high‐sensitivity cardiac troponin T (hs‐cTnT) was an independent predictor of 30‐day mortality. Using an age‐adjusted threshold (≥ 14 pg/mL for patients < 75 years and ≥ 45 pg/mL for those ≥ 75 years), elevated troponin was associated with an odds ratio of 3.38 (95% CI: 1.33–8.58, *p* = 0.010) [37]. Moreover, the role of troponin in predicting long‐term mortality was demonstrated by Lee Chuy et al., who found that patients with detectable high‐sensitivity cardiac troponin I (hs‐cTnI) had a higher all‐cause mortality rate (29%) compared to those with undetectable levels (20%, *p* = 0.05) [64]. Overall, the findings from studies not included in the quantitative analysis were largely consistent with the pooled results.

### 3.4 ICU Admission

Along with its association with increased mortality, elevated troponin levels have also been linked to a higher incidence of complications requiring ICU admission. Several studies included in the review evaluated the relationship between troponin elevation and ICU admission, reinforcing its prognostic value in acute PE. For instance, Goraya et al. examined the predictive value of hs‐cTnT for ICU admission and found a strong association: patients with hs‐cTnT ≥ 12 pg/mL had significantly higher odds of ICU admission (OR: 13.8; 95% CI: 5.38–35.4). This association remained robust after adjusting for sPESI (Simplified Pulmonary Embolism Severity Index) score and serum creatinine (adjusted OR: 14.5; 95% CI: 5.05–41.5) [65]. The study also identified an optimal cutoff of hs‐cTnT (≥ 12 pg/mL), which demonstrated high sensitivity (93.5%) and negative predictive value (95.9%) for ruling out ICU admission. Similarly, Hakemi et al. reported that ICU admission was significantly less frequent among patients with undetectable hs‐TnI. Only 9% of patients with undetectable hs‐cTnI required ICU‐level care compared to 38% of those with elevated levels (*p* < 0.001) [63]. This pattern held for both “hard” ICU admissions (e.g., related to haemodynamic instability) and “soft” admissions (e.g., precautionary monitoring), highlighting the potential utility of undetectable troponin as a negative predictor for escalation of care. Moreover, Ng et al. demonstrated a similar trend using a standard‐sensitivity cTnI assay (Access AccuTnI, Beckman Coulter). In their study, patients with cTnI levels greater than 0.04 ng/mL were nearly four times more likely to require ICU admission compared to those with normal cTnI levels (OR: 3.9; 95% CI: 1.1–14.2; *p* = 0.031) [48]. Overall, these findings suggest that elevated troponin levels are strong predictors of ICU admission in patients with acute PE, supporting their potential role in early risk stratification and guiding decisions regarding the intensity of monitoring and care.

### 3.5 Haemodynamic Instability

Haemodynamic instability is a critical indicator of disease severity in acute PE, with several studies evaluating the association between elevated troponin levels and these adverse clinical features, highlighting the utility of troponin in identifying high‐risk patients. A study by Kim et al. [52] demonstrated that hs‐cTnT was significantly associated with clinical markers of haemodynamic compromise, including hypotension and shock. Patients classified as high‐risk based on a systolic blood pressure < 90 mmHg and/or the presence of shock exhibited substantially elevated hs‐cTnT levels compared to normotensive patients [52]. Similarly, Kukla et al. investigated the prognostic value of troponin among intermediate‐risk PE patients—defined according to European Society of Cardiology (ESC) guidelines as individuals without persistent hypotension or shock, but with evidence of RVD and/or elevated cardiac troponin indicating myocardial injury. Among this subgroup, 60% had elevated troponin, which was significantly associated with an increased risk of complications, including cardiogenic shock, vasopressor requirement, and ventilatory support (OR: 4.94; 95% CI: 1.63–22.3; *p* = 0.003) [34]. In addition, Aksay et al. reported that elevated cTnI (≥ 0.5 ng/mL) was significantly associated with hypotension at emergency department presentation. Among patients with elevated cTnI, 48.5% experienced hypotension compared to only 11.4% of those with normal cTnI, corresponding to an odds ratio of 7.34 (95% CI: 2.31–23.28; *p* < 0.001) [13]. These findings suggest that elevated troponin levels are strongly associated with haemodynamic instability, particularly hypotension and shock, underscoring their value in early risk stratification and identification of patients at risk for clinical deterioration.

### 3.6 RVD

As a marker of cardiac injury, troponin elevation in acute PE is often a reflection of RVD. Aksay et al. reported that among cTnI‐positive patients who underwent echocardiography, 92% demonstrated RVD compared to only 19% of those with normal cTnI levels (*p* < 0.001) [13]. Common echocardiographic abnormalities in the elevated cTnI group included right ventricular dilation (96%), paradoxical septal motion (43%), and RV hypokinesia (39%), indicating a strong correlation between biochemical myocardial injury and imaging‐confirmed RV impairment. These findings suggest that cTnI elevation in PE likely reflects increased RV afterload and mechanical strain, reinforcing its utility in risk stratification. Similarly, La Vecchia et al. found that patients with elevated cTnI more frequently exhibited echocardiographic and clinical markers of RVD, including RV dilatation and hypokinesia, elevated pulmonary artery pressures, and greater central pulmonary artery involvement on imaging. These consistent observations support the use of troponin not only as a mortality marker but also as a surrogate for structural and functional RV compromise.

## 4 Discussion

This review analysed 60 studies, incorporating data from a total of 25,282 patients. We conducted five meta‐analyses to evaluate 30‐day all‐cause mortality, in‐hospital all‐cause mortality, RVD, haemodynamic instability, and ICU admission. All five analyses revealed a significant association between troponin elevation and the adverse outcome. We also conducted a narrative synthesis of these outcomes, which reported similar findings to the quantitative synthesis. These findings support the prognostic value of troponin in PE. Publication bias was assessed using funnel plots and Egger's regression test. Evidence of funnel plot asymmetry was observed for mortality outcomes, with Egger's test confirming significant small‐study effects for both in‐hospital and 30‐day mortality, suggesting the presence of publication bias. In contrast, Egger's test did not demonstrate considerable asymmetry for RVD, hemodynamic instability, or ICU admission, indicating no strong evidence of publication bias for these outcomes. Taken together, while publication bias may have influenced the mortality analyses, the consistency and magnitude of the associations across large, high‐quality studies support the robustness of our overall findings.

Troponin elevation likely reflects RV myocardial strain caused by increased afterload from pulmonary arterial obstruction, contributing to worse clinical outcomes. Importantly, several included studies demonstrated that even normotensive or “low‐risk” patients with elevated troponin had significantly poorer outcomes, indicating that its prognostic value extends beyond traditionally defined high‐risk patients. Troponin‐based risk stratification may therefore enable proactive identification of patients at risk of decompensation, facilitating earlier escalation of care and potentially improving clinical outcomes and reducing mortality in PE. In the emergency setting, troponin measurement may also aid in disposition planning. Patients with elevated troponin levels may benefit from more intensive monitoring or early ICU admission, even in the absence of overt haemodynamic instability. Conversely, the absence of troponin elevation in clinically stable patients may support ward‐level care or, in selected low‐risk individuals, consideration for early discharge and outpatient management.

Our study also evaluated prognostic utility across various assay types, including both conventional and high‐sensitivity troponin assays, and found that results were broadly consistent regardless of the specific assay used. This supports the applicability of troponin as a prognostic marker across various clinical settings and testing platforms.

Our findings are consistent with prior systematic reviews. A 2018 review by Darwish et al. focused on the prognostic value of troponin in low‐risk PE patients and found a significant association with short‐term adverse outcomes [66]. Similarly, a 2020 review by Nithianandan et al. assessed troponin's predictive role after applying specific clinical eligibility criteria and supported its prognostic utility in select patient subgroups [67]. Bajaj et al. [68] examined patients with acute non‐massive PE and also found that elevated troponin levels were significantly associated with increased short‐term mortality risk [68]. Similar to Darwish et al., Barco et al. [68] also demonstrated that elevated troponin levels predicted mortality among patients classified as low‐risk, further reinforcing its role in early risk stratification [69].

Compared to these studies, our review encompasses a larger number of studies, incorporates more recent publications, and provides a comprehensive analysis across high‐, intermediate‐, and low‐risk PE populations. To our knowledge, this is the most up‐to‐date and thorough synthesis of the prognostic value of troponin in acute PE. Moreover, our review evaluates multiple clinical outcomes beyond mortality, including ICU admission, haemodynamic instability, and RVD. These additions provide a more nuanced and clinically relevant understanding of troponin's role in the risk stratification of PE.

This study has several strengths. These include a large pooled patient sample, a dual quantitative synthesis of two distinct mortality outcomes (30‐day and in‐hospital), and an in‐depth evaluation of a wide spectrum of PE presentations using different troponin assay types. Furthermore, the certainty of evidence was high for mortality and haemodynamic outcomes, while moderate for RVD and ICU admission. However, certain limitations must be acknowledged. Heterogeneity in assay type, variable cut‐off thresholds, and differences in the timing of troponin measurements may affect the generalisability of our findings. Evidence of publication bias was observed for the mortality outcomes, as indicated by significant funnel plot asymmetry and Egger's test, which may have influenced the strength of these associations.

Furthermore, our analysis was limited to English‐language studies, which may also affect generalisability. Additionally, some studies did not provide data suitable for meta‐analysis and were therefore excluded from the quantitative synthesis. For 30‐day mortality, ICU admission, and RVD, heterogeneity in reporting resulted in the exclusion of two studies each for 30‐day mortality and ICU admission, and four studies for RVD. There were few studies which provided sufficient data for ICU admission, and therefore, only four studies were included in the quantitative synthesis. Moreover, we were unable to stratify outcomes by severity, as the majority of studies did not report data in this manner. It is also important to acknowledge that elevated troponin levels may result from conditions unrelated to PE. Comorbidities such as chronic kidney disease, heart failure, or sepsis can independently elevate troponin, potentially confounding risk stratification. Therefore, troponin levels should always be interpreted in the context of the broader clinical picture, ideally in conjunction with imaging and clinical scoring systems.

Future research should aim to identify optimal cut‐off values for prognostically significant troponin elevations, as relatively few studies have addressed this issue. Additionally, future analyses should stratify findings by assay type (e.g., troponin I vs. T, conventional vs. high‐sensitivity) to clarify which assays provide the most reliable prognostic information. Furthermore, outcomes should be stratified by severity to allow for more nuanced analyses and to better understand whether the predictive value of troponin varies across different levels of disease severity. Finally, prospective studies are needed to explore troponin's role in risk‐stratification models, particularly in guiding decisions around early ICU admission, vasopressor initiation, or advanced therapeutic interventions. These studies should focus on the accuracy and predictive performance of troponin, rather than merely its correlation with outcomes, to fully evaluate its role in clinical decision‐making.

## 5 Conclusion

This review highlights the strong association between troponin elevation and adverse prognostic outcomes in patients with acute PE. Troponin elevation was consistently linked not only to increased short‐term mortality but also to clinical deterioration indicators such as ICU admission, hypotension, and RVD. To fully realize its potential, future research should focus on validating troponin‐guided management strategies in prospective studies, particularly in normotensive and intermediate‐risk populations. Standardization of assay type and cutoff thresholds will also be essential to improve consistency in clinical applications.

## Conflicts of Interest

The authors declare no conflicts of interest.

## Supporting information


**S1:** Forest plot of studies assessing the association between elevated troponin levels and in‐hospital all‐cause mortality in patients with acute pulmonary embolism. **S2:** Forest plot of studies assessing the association between elevated troponin levels and 30‐day all‐cause mortality in patients with acute pulmonary embolism. **S3:** Forest plot of studies assessing the association between elevated troponin levels and right ventricular dysfunction (RVD) in patients with acute pulmonary embolism. **S4:** Forest plot of studies assessing the association between elevated troponin levels and ICU admission in patients with acute pulmonary embolism. **S5:** Forest plot of studies assessing the association between elevated troponin levels and haemodynamic instability in patients with acute pulmonary embolism. **S6:** Funnel plot of included studies assessing the association between elevated troponin and in‐hospital all‐cause mortality. The plot demonstrates asymmetry, supported by Egger's regression test. **S7:** Funnel plot assessing potential publication bias for studies evaluating the association between troponin elevation and 30‐day all‐cause mortality. The plot demonstrates asymmetry, supported by Egger's regression test. **S8:** Funnel plot assessing potential publication bias for studies evaluating the association between troponin elevation and right ventricular dysfunction (RVD). The plot does not show significant asymmetry, supported by Egger's regression test. **S9:** Funnel plot assessing potential publication bias for studies evaluating the association between troponin elevation and haemodynamic instability. The plot does not show significant asymmetry, supported by Egger's regression test. **S10:** Funnel plot assessing potential publication bias for studies evaluating the association between troponin elevation and ICU admission. The plot does not show significant asymmetry, supported by Egger's regression test.

## Data Availability

Data relating to this study are available upon reasonable request from the corresponding author.
