# Supplementary material for: The Prognostic Value of Troponin in Acute Pulmonary Embolism: A Systematic Review and Meta‐Analysis
Source: Catheter Cardiovasc Interv. 2025 Oct 21;106(7):3899–916. doi: 10.1002/ccd.70243 (PMC12679527; doi:10.1002/ccd.70243)
Supplement: Supplementary file 1 — S1: Forest plot of studies assessing the association between elevated troponin levels and in‐hospital all‐cause mortality in patients with acute pulmonary embolism. S2: Forest plot of studies assessing the association between elevated troponin levels and 30‐day all‐cause mortality in patients with acute pulmonary embolism. S3: Forest plot of studies assessing the association between elevated troponin levels and right ventricular dysfunction (RVD) in patients with acute pulmonary embolism. S4: Forest plot of studies assessing the association between elevated troponin levels and ICU admission in patients with acute pulmonary embolism. S5: Forest plot of studies assessing the association between elevated troponin levels and haemodynamic instability in patients with acute pulmonary embolism. S6: Funnel plot of included studies assessing the association between elevated troponin and in‐hospital all‐cause mortality. The plot demonstrates asymmetry, supported by Egger's regression test. S7: Funnel plot assessing potential publication bias for studies evaluating the association between troponin elevation and 30‐day all‐cause mortality. The plot demonstrates asymmetry, supported by Egger's regression test. S8: Funnel plot assessing potential publication bias for studies evaluating the association between troponin elevation and right ventricular dysfunction (RVD). The plot does not show significant asymmetry, supported by Egger's regression test. S9: Funnel plot assessing potential publication bias for studies evaluating the association between troponin elevation and haemodynamic instability. The plot does not show significant asymmetry, supported by Egger's regression test. S10: Funnel plot assessing potential publication bias for studies evaluating the association between troponin elevation and ICU admission. The plot does not show significant asymmetry, supported by Egger's regression test. [file CCD-106-3899-s001.docx]

S1: Forest plot of studies assessing the association between elevated troponin levels and in-hospital all-cause mortality in patients with acute pulmonary embolism.

**
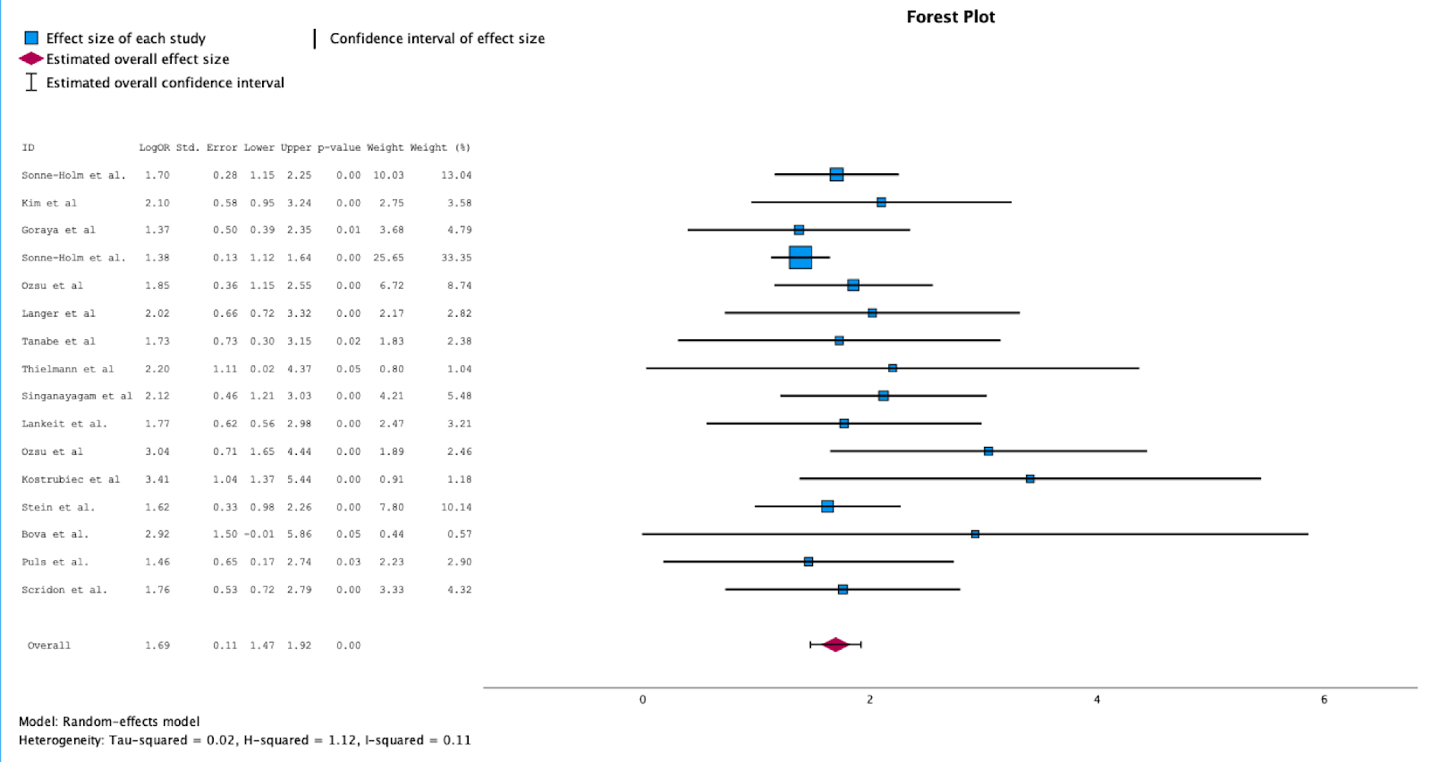
**

S2: Forest plot of studies assessing the association between elevated troponin levels and 30-day all-cause mortality in patients with acute pulmonary embolism.

**
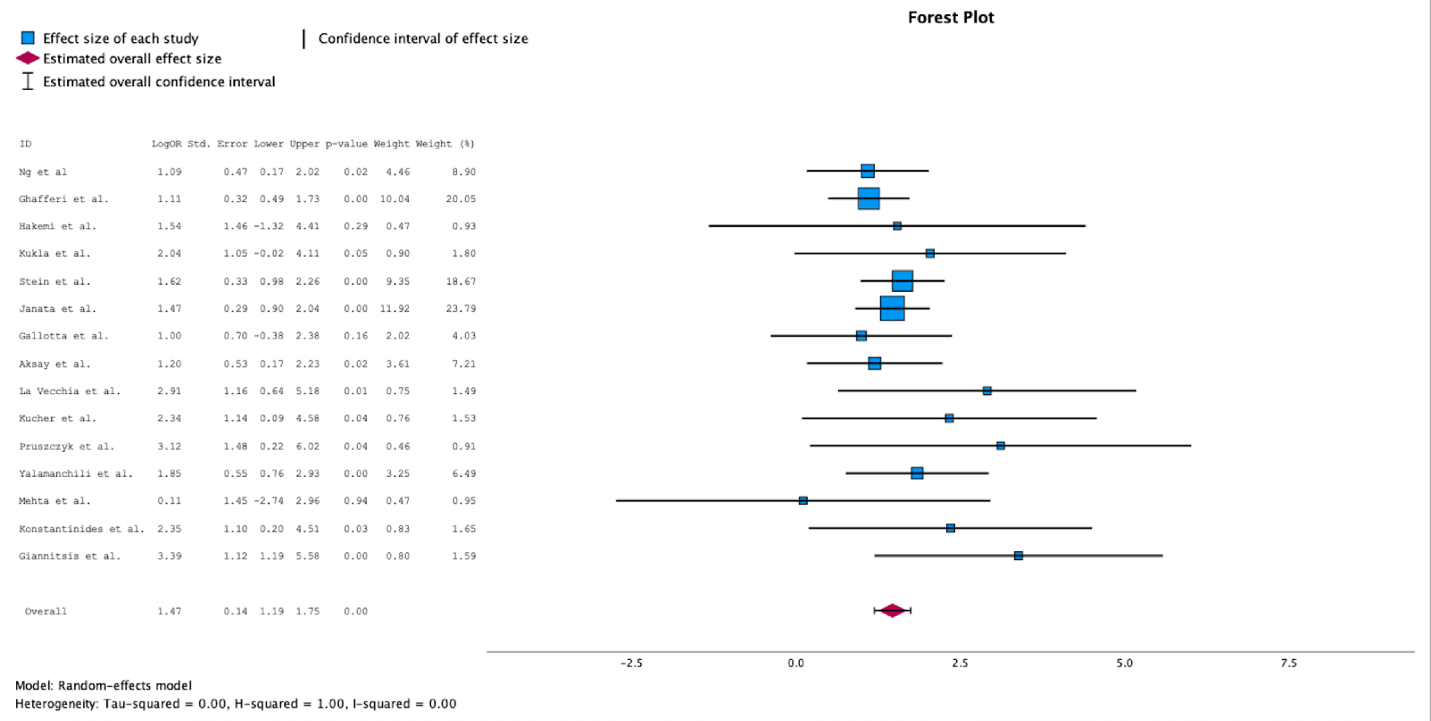
**


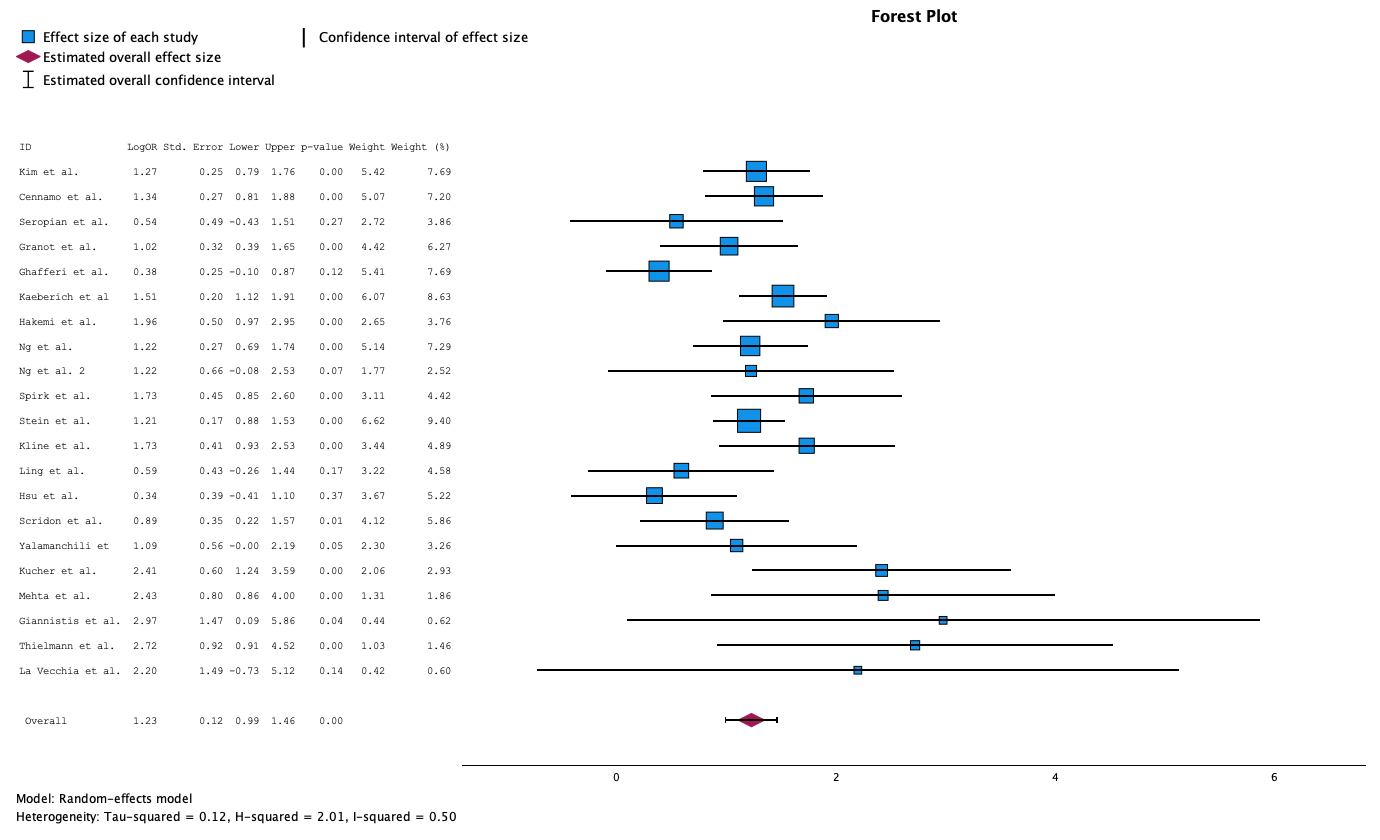


S3: Forest plot of studies assessing the association between elevated troponin levels and right ventricular dysfunction in patients with acute pulmonary embolism.


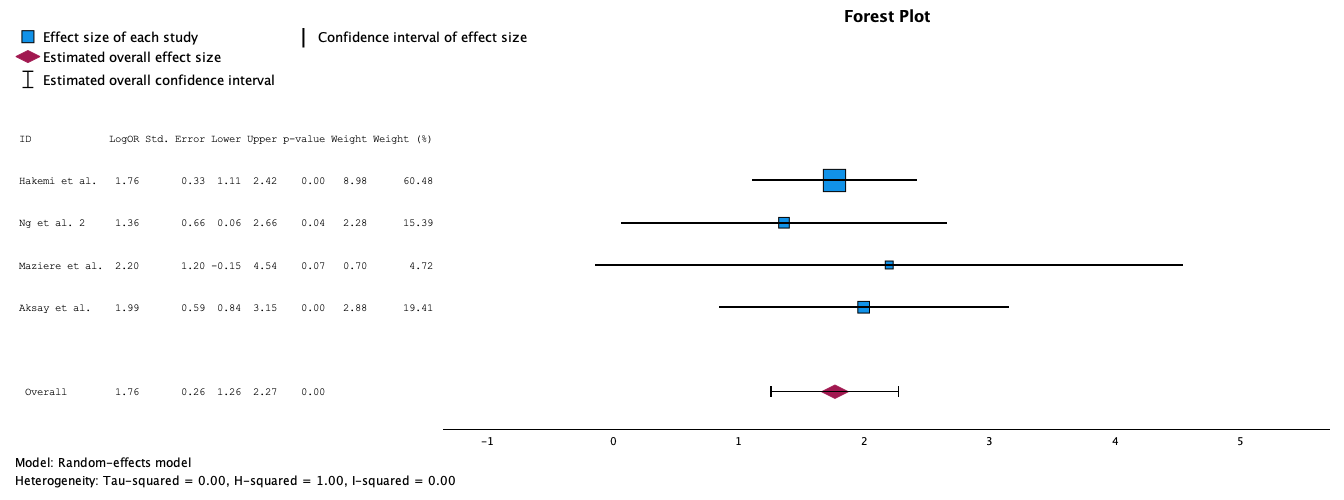


S4: Forest plot of studies assessing the association between elevated troponin levels and ICU admission in patients with acute pulmonary embolism.


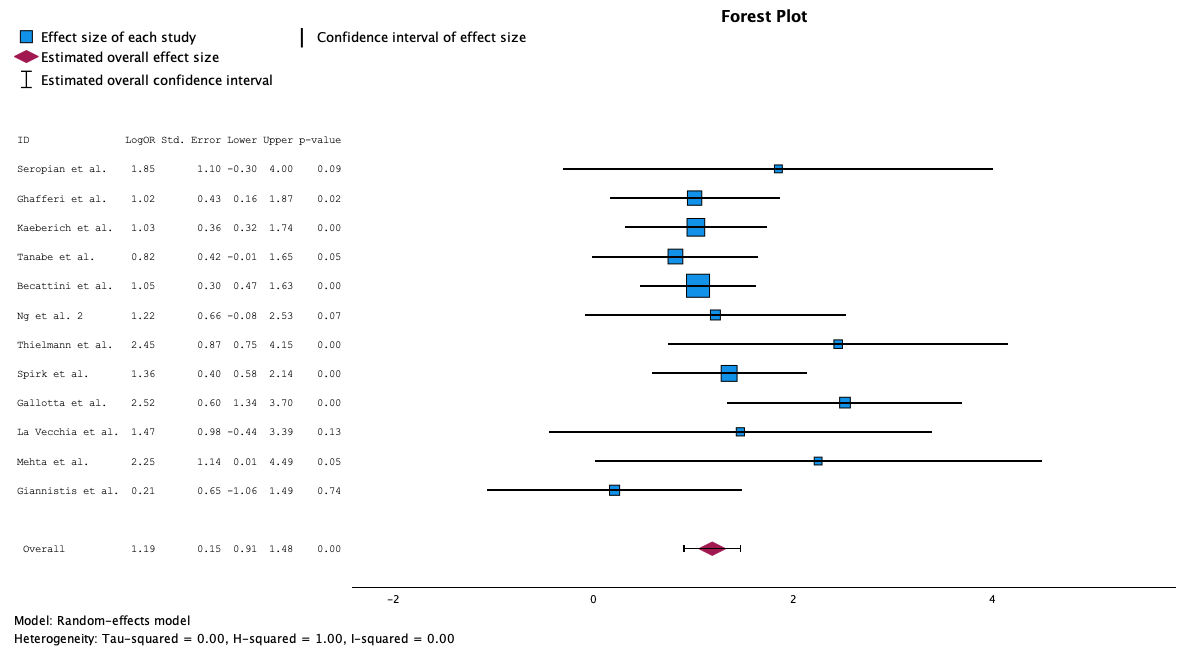


S5: Forest plot of studies assessing the association between elevated troponin levels and haemodynamic instability in patients with acute pulmonary embolism.


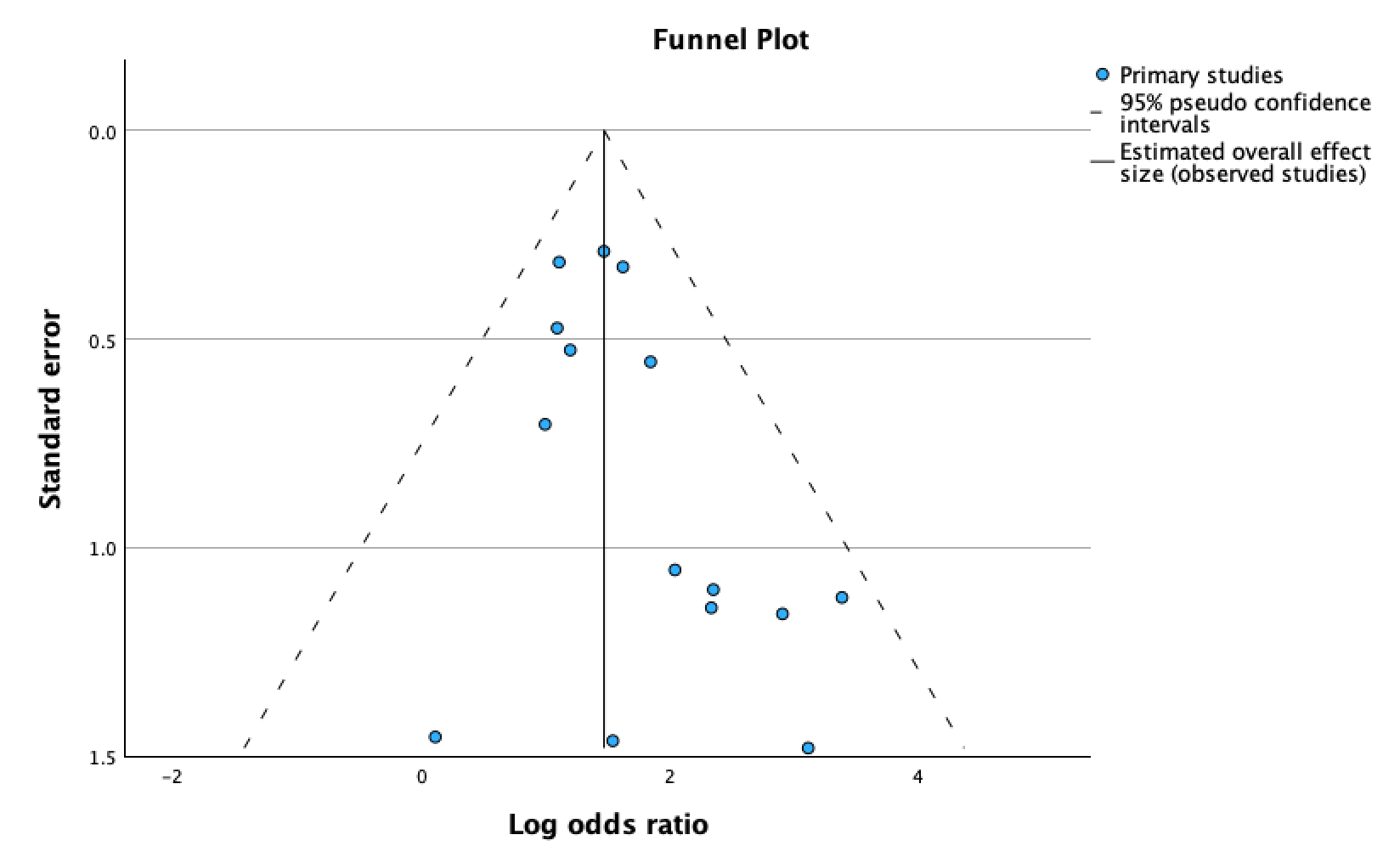


| **Egger's Regression-Based Test^a^** | | | | | | |
| --- | --- | --- | --- | --- | --- | --- |
| Parameter | Coefficient | Std. Error | t | Sig. (2-tailed) | 95% Confidence Interval | |
|  |  |  |  |  | Lower | Upper |
| (Intercept) | 1.241 | .1583 | 7.840 | <.001 | .902 | 1.581 |
| SE^b^ | 1.238 | .4246 | 2.915 | .011 | .327 | 2.148 |
| a. Random-effects meta-regression | | | | | | |
| b. Standard error of effect size | | | | | | |

S6: Funnel plot of included studies assessing the association between elevated troponin and in-hospital all-cause mortality. The plot demonstrates asymmetry, supported by Egger’s regression test.


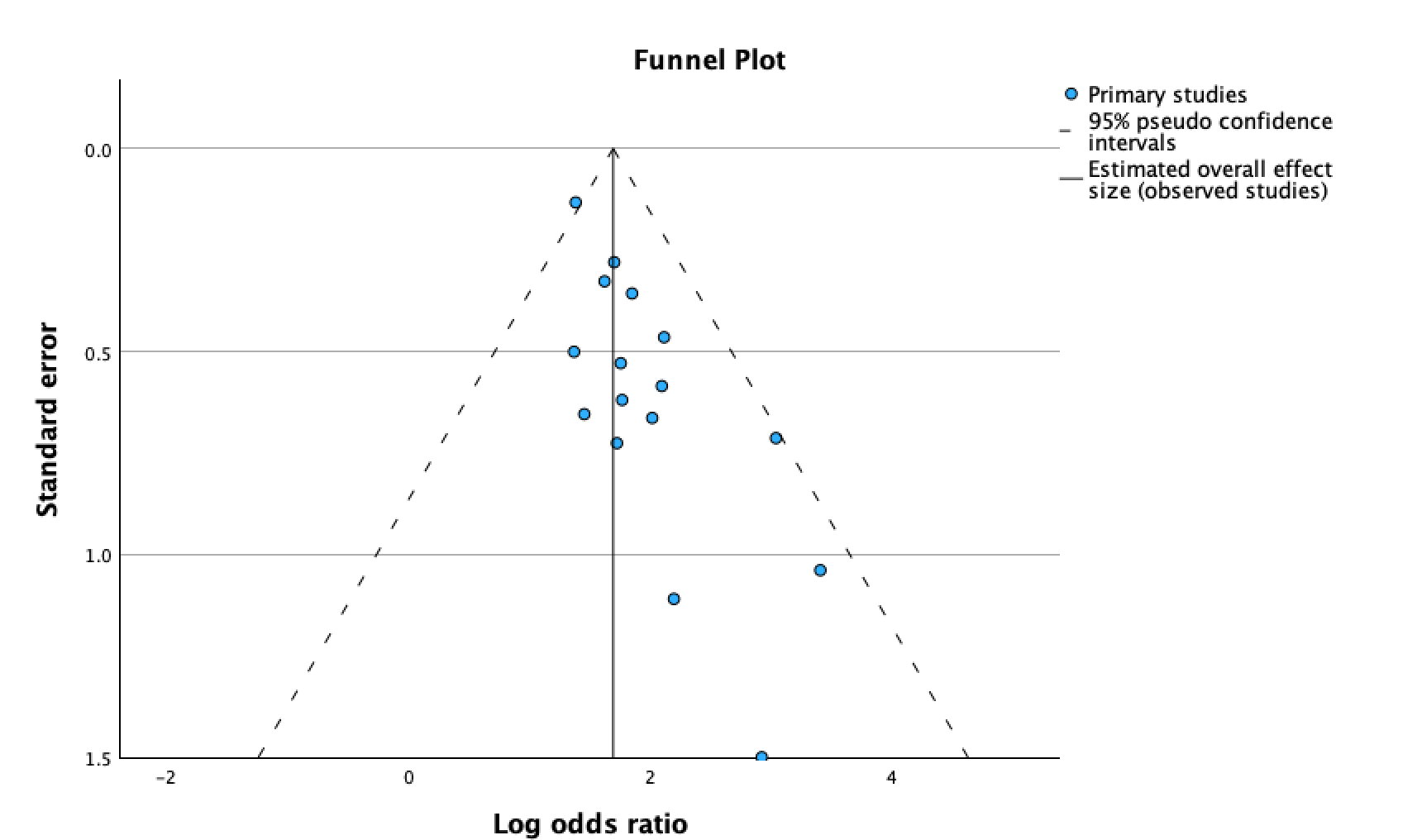


| **Egger's Regression-Based Test^a^** | | | | | | |
| --- | --- | --- | --- | --- | --- | --- |
| Parameter | Coefficient | Std. Error | t | Sig. (2-tailed) | 95% Confidence Interval | |
|  |  |  |  |  | Lower | Upper |
| (Intercept) | 1.241 | .1583 | 7.840 | <.001 | .902 | 1.581 |
| SE^b^ | 1.238 | .4246 | 2.915 | .011 | .327 | 2.148 |
| a. Random-effects meta-regression | | | | | | |
| b. Standard error of effect size | | | | | | |

S7: Funnel plot assessing potential publication bias for studies evaluating the association between troponin elevation and 30-day all-cause mortality. The plot demonstrates asymmetry, supported by Egger’s regression test.


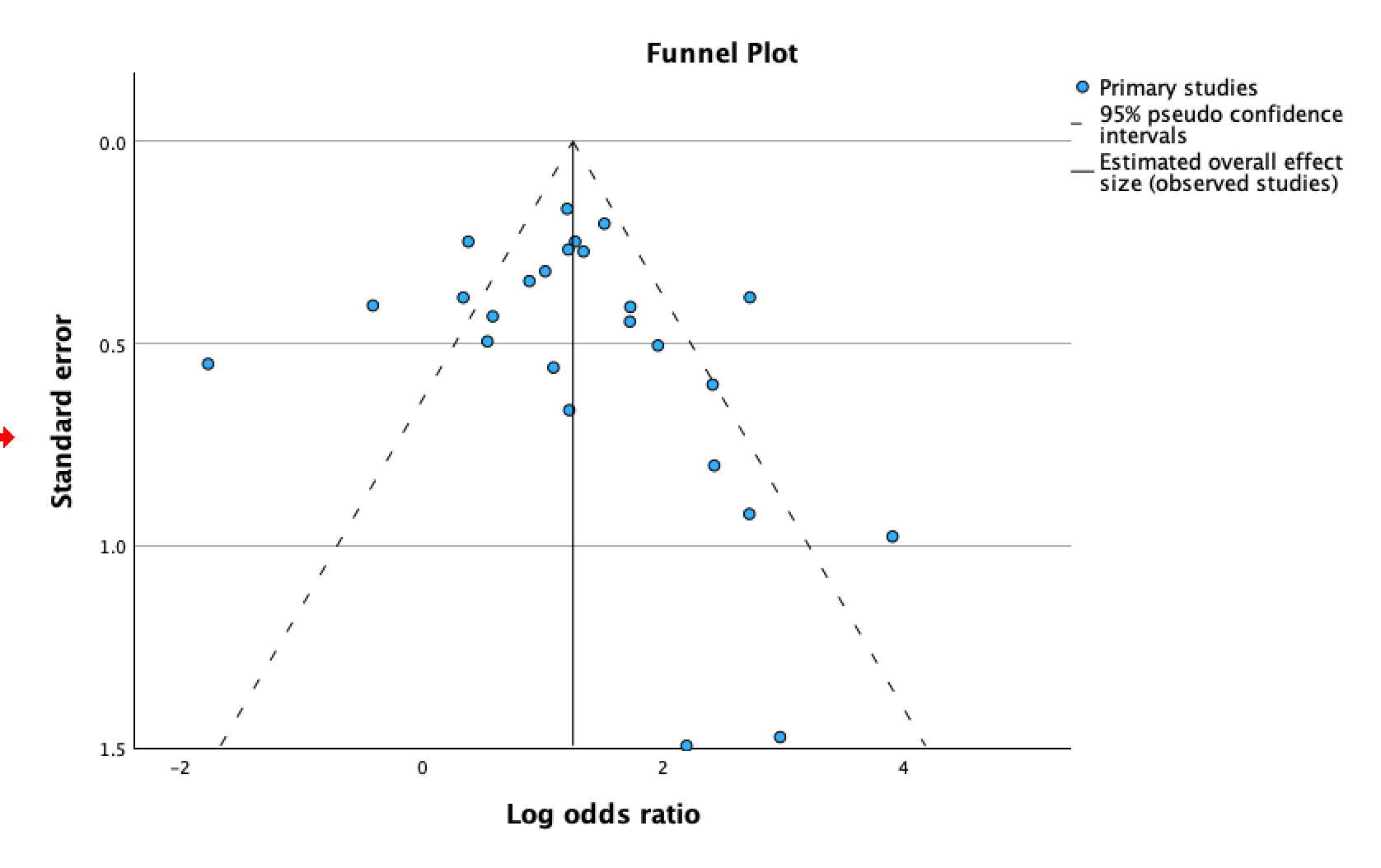


| **Egger's Regression-Based Test^a^** | | | | | | |
| --- | --- | --- | --- | --- | --- | --- |
| Parameter | Coefficient | Std. Error | t | Sig. (2-tailed) | 95% Confidence Interval | |
|  |  |  |  |  | Lower | Upper |
| (Intercept) | .525 | .4072 | 1.288 | .211 | -.318 | 1.367 |
| SE^b^ | 1.608 | .7866 | 2.044 | .053 | -.019 | 3.235 |
| a. Random-effects meta-regression | | | | | | |
| b. Standard error of effect size | | | | | | |

S8: Funnel plot assessing potential publication bias for studies evaluating the association between troponin elevation and RVD. The plot does not show significant asymmetry, supported by Egger’s regression test.


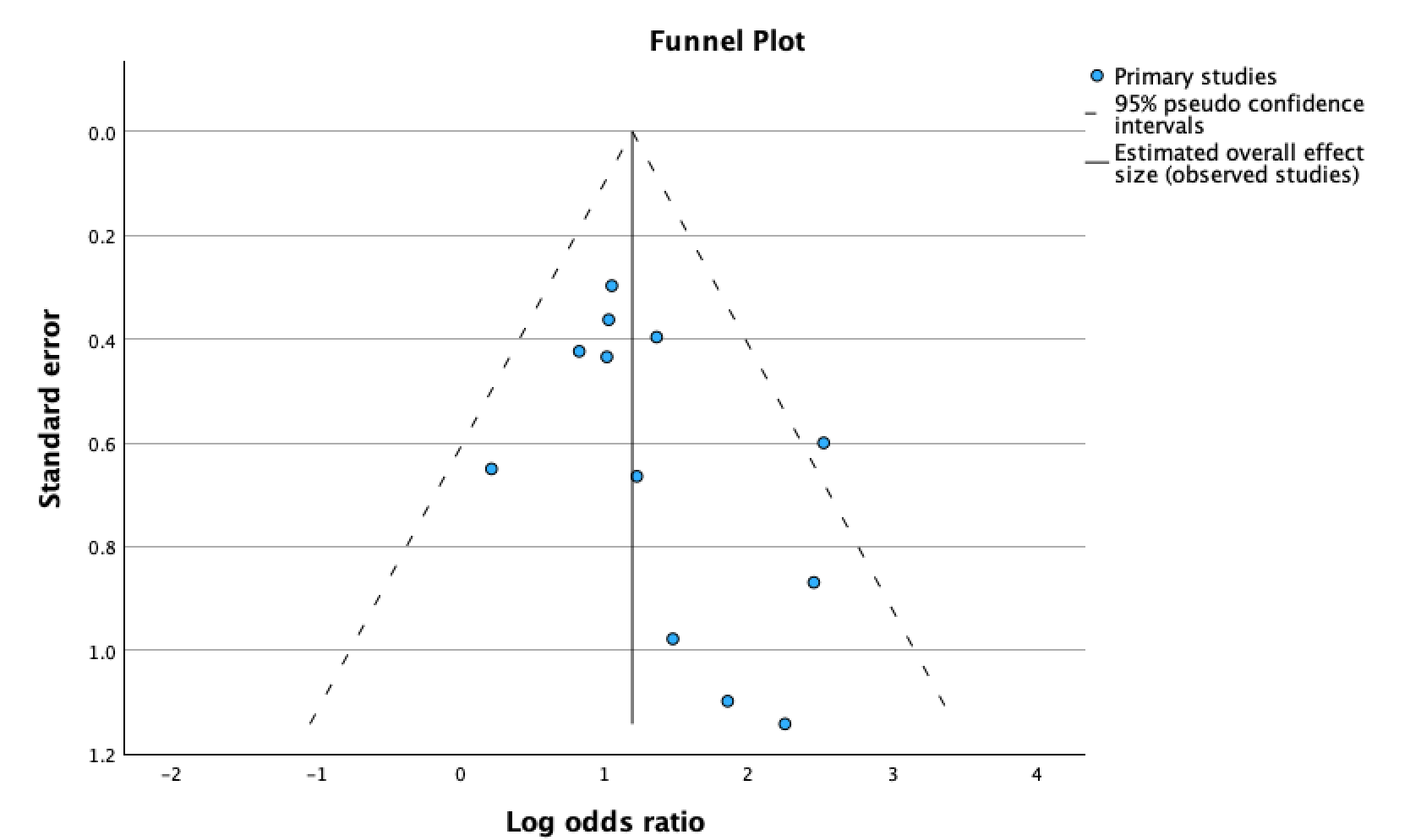


| **Egger's Regression-Based Test^a^** | | | | | | |
| --- | --- | --- | --- | --- | --- | --- |
| Parameter | Coefficient | Std. Error | t | Sig. (2-tailed) | 95% Confidence Interval | |
|  |  |  |  |  | Lower | Upper |
| (Intercept) | .628 | .3704 | 1.695 | .121 | -.198 | 1.453 |
| SE^b^ | 1.221 | .7375 | 1.655 | .129 | -.423 | 2.864 |
| a. Random-effects meta-regression | | | | | | |
| b. Standard error of effect size | | | | | | |

S9: Funnel plot assessing potential publication bias for studies evaluating the association between troponin elevation and haemodynamic instability. The plot does not show significant asymmetry, supported by Egger’s regression test.


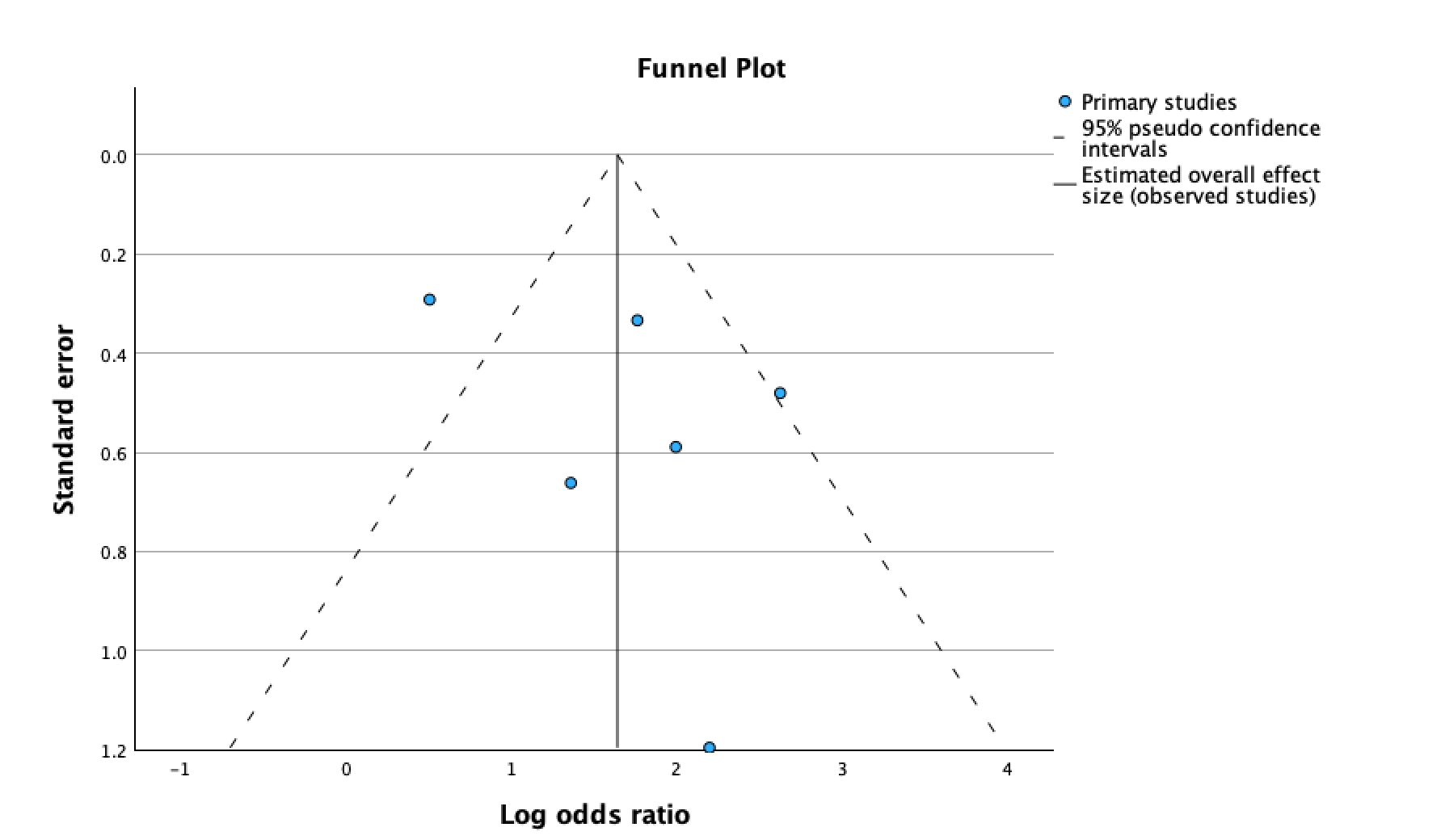


| **Egger's Regression-Based Test^a^** | | | | | | |
| --- | --- | --- | --- | --- | --- | --- |
| Parameter | Coefficient | Std. Error | t | Sig. (2-tailed) | 95% Confidence Interval | |
|  |  |  |  |  | Lower | Upper |
| (Intercept) | .975 | .8347 | 1.168 | .308 | -1.343 | 3.292 |
| SE^b^ | 1.338 | 1.5313 | .874 | .432 | -2.914 | 5.589 |
| a. Random-effects meta-regression | | | | | | |
| b. Standard error of effect size | | | | | | |

S10: Funnel plot assessing potential publication bias for studies evaluating the association between troponin elevation ICU admission. The plot does not show significant asymmetry, supported by Egger’s regression test.
